# Review: The Role of Intestinal Dysbiosis in Parkinson’s Disease

**DOI:** 10.3389/fcimb.2021.615075

**Published:** 2021-04-22

**Authors:** Yiying Huang, Jinchi Liao, Xu Liu, Yunxiao Zhong, Xiaodong Cai, Ling Long

**Affiliations:** ^1^ Department of Neurology, Third Affiliated Hospital, Sun Yat-Sen University, Guangzhou, China; ^2^ Department of Neurology, Sixth Affiliated Hospital, Sun Yat-Sen University (Guangdong Gastrointestinal and Anal Hospital), Guangzhou, China

**Keywords:** Parkinson’s disease, intestinal dysbiosis, α-synuclein, inflammation, molecular mimicry

## Abstract

Several studies have highlighted the roles played by the gut microbiome in central nervous system diseases. Clinical symptoms and neuropathology have suggested that Parkinson’s disease may originate in the gut, which is home to approximately 100 trillion microbes. Alterations in the gastrointestinal microbiota populations may promote the development and progression of Parkinson’s disease. Here, we reviewed existing studies that have explored the role of intestinal dysbiosis in Parkinson’s disease, focusing on the roles of microbiota, their metabolites, and components in inflammation, barrier failure, microglial activation, and α-synuclein pathology. We conclude that there are intestinal dysbiosis in Parkinson’s disease. Intestinal dysbiosis is likely involved in the pathogenesis of Parkinson’s disease through mechanisms that include barrier destruction, inflammation and oxidative stress, decreased dopamine production, and molecular mimicry. Additional studies remain necessary to explore and verify the mechanisms through which dysbiosis may cause or promote Parkinson’s disease. Preclinical studies have shown that gastrointestinal microbial therapy may represent an effective and novel treatment for Parkinson’s disease; however, more studies, especially clinical studies, are necessary to explore the curative effects of microbial therapy in Parkinson’s disease.

## Introduction

Parkinson’s disease (PD) is the second-most common neurodegenerative disease, after Alzheimer’s disease. Approximately seven to ten million PD patients have been diagnosed worldwide (Nair et al., 2018), and this population is expected to double by 2030 (Dorsey et al., 2007). PD primarily presents as motor dysfunction including tremor, rigidity, postural instability, and bradykinesia. The non-motor symptoms of PD include anosmia, sleep disturbance, anxiety, depression, orthostatic hypotension, and gastrointestinal (GI) symptoms. GI symptoms such as dyspepsia, hypersalivation, constipation, defecatory dysfunction, fecal incontinence, nausea, and abdominal pain usually precede motor symptoms (Cersosimo et al., 2013; Mukherjee et al., 2016). Constipation is a major intestinal dysfunction associated with PD and represents one of the earliest biomarkers of the pathologic process that ultimately results in PD development (Kalia and Lang, 2015). Constipation and intestinal inflammation often occur years before the onset of motor symptoms (Braak et al., 2003; Verbaan et al., 2007; Devos et al., 2013; Kalia and Lang, 2015). The primary pathological features of PD include the progressive loss of dopaminergic neurons in the substantia nigra (SN) and the formation of cytoplasmic inclusions comprising abnormal, aggregating α-synuclein (αSyn) proteins known as Lewy bodies. αSyn oligomers are deposited not only in the SN but also in the brainstem, olfactory bulb, and GI tract (Punsoni et al., 2019), and Braak hypothesized that abnormal αSyn accumulates in the gut and spreads to the brain in a prion-like manner through the vagus nerve (Del Tredici and Braak, 2008). This view is supported by pathophysiological evidence that revealed the early appearance of αSyn-containing inclusion bodies in the enteric nervous system (ENS), the glossopharyngeal nerve, and the vagus nerve (Braak et al., 2003; Shannon et al., 2012), and the risk of PD development was shown to be reduced among individuals with vagus nerve amputations (Svensson et al., 2015; Liu et al., 2017). Recent studies have further confirmed Braak’s proposal. When PFFs (the preformed αSyn fibrils)were injected into the myenteric plexus of pylorus and duodenal, pSer129-αSyn (phosphorylated on serine 129-αSyn) immunoreactivity could be detected in wild-type mice and BAC (Bacterial Artificial Chromosome) rats (the transgenic rat model overexpresses the complete human SNCA gene in its wild-type form) at the original injection site, dorsal motor nucleus of the vagus nerve and substantia nigra pars compacta, while it could only be detected in truncal vagotomy animals at the original injection site, but not in substantia nigra (Kim et al., 2019; Van Den Berge et al., 2019). Because PD appears to display pathological and clinical manifestations of the GI tract earlier than typical PD symptoms and severing the connections between the GI tract and the central nervous system (CNS) has been shown to reduce PD risk, it’s reasonable to assume that local factors in the GI tract may contribute to PD development. With an area half the size of a badminton field (Helander and Fandriks, 2014), the digestive tract is susceptible to intestinal dysbiosis, as the digestive tract serves as the body’s primary microorganism reservoir (Sender et al., 2016b). This dysbiosis may lead to local inflammation, pathological changes, and intestinal symptoms.

## Intestinal Dysbiosis and Brain Health

The majority of the human microbiome is concentrated in the gut, which contains more than 10 trillion cells and up to 1,000 different microbial species (Sender et al., 2016a). The term “microbiome” refers to the genetic material (genome) contained in all microbiota found in the GI system, representing a collection of bacteria, viruses, archaea, fungi, and protozoa (Lozupone et al., 2012). Gut microbiota is a general term for all symbionts living in the gut (Adak and Khan, 2019). Most studies to date examining intestinal microorganisms focused on intestinal bacteria. Intestinal bacteria are mainly divided into seven branches, including Firmicutes, Bacteroidetes, Actinobacteria, Fusobacteria, Proteobacteria, Verrucomicrobia and Cyanobacteria, and the number of Bacteroidetes and Firmicutes accounts for more than 90% (Adak and Khan, 2019). Intestinal dysbiosis is defined as the disruption of the “normal” balance between GI microbiota and the host (Lozupone et al., 2012). Intestinal dysbiosis has been associated with obesity (Turnbaugh et al., 2008; Just et al., 2018), malnutrition (Ghosh et al., 2014), diabetes (Giongo et al., 2011; Karlsson et al., 2013), cardiovascular disease (Cho et al., 2017), autistic spectrum disorders (Parracho et al., 2005) and Alzheimer’s disease (Vogt et al., 2017; Sochocka et al., 2019). In recent decades, a deeper understanding of GI microbiota populations has greatly complemented the concept of the gut-brain axis, which has evolved into the concept of the microbiota-gut-brain axis. Microbiota and the brain communicate with each other *via* various routes including the immune system, tryptophan metabolism, the vagus nerve, and the ENS through microbial metabolites such as short-chain fatty acids (SCFAs), branched-chain amino acids, and peptidoglycans (Cryan et al., 2019). GI microbiota are essential for maintaining a healthy nervous system. On the one hand, GI microbiota play roles in basic neurogenerative processes such as the formation of the blood–brain–barrier (BBB), myelination, neurogenesis, and microglia maturation (Sharon et al., 2016), as demonstrated by germ-free (GF) mice, which develop abnormal hypothalamic-pituitary-adrenal axes, changes in hippocampal nerves, reduced mRNA and protein levels of hippocampal brain-derived nerve growth factor (BNGF), and impaired BBB function (Sudo et al., 2004; Braniste et al., 2014; Mohle et al., 2016). On the other hand, GI microbiota are highly important to the immune system (Rooks and Garrett, 2016), as they control microglial maturation and its function (Erny et al., 2015), and GF mice show immunodeficiencies at both the cellular and structural levels (Round and Mazmanian, 2009). When colonizing human bodies, various microbes provide an abundance of antigens, which are essential for the healthy maturation of the immune system during early life (Cahenzli et al., 2013). It is worth emphasizing that microbial homeostasis is crucial to individual health.

It has been proved that intestinal dysbiosis can promote the pathology of PD. Compared with mice exposed to lipopolysaccharide (LPS) negative bacteria, mice injected with αSyn and exposed to LPS positive bacteria produced a unique form of αSyn fibers. This abnormal protein aggregation may further form the typical pathological changes of PD (Fitzgerald et al., 2019). In another study, the mRNA level of αSyn in SH-SY5Y cells (human neuroblastoma) treated with B. mirabilis was significantly higher than that in the untreated cells. This means that there may be an increase in the production of αSyn. They further investigated the differences in the aggregation of αSyn: on day 16 after administration of M. mirabilis, αSyn filaments in the distal colon increased significantly, and in the SN, the immunoreactivity of αSyn filaments was significantly stronger than that of normal group (Choi et al., 2018).

## Altered Gastrointestinal Microbiota Composition in PD

Intestinal dysbiosis has been proposed to act as a trigger or enabler of PD. In mice that overexpress αSyn, GI microbiota aggravates the expression of motor deficits, microglial activation, and α-Syn pathology. The deficits reduce but don’t disappear entirely in the absence of microbiota (Sampson et al., 2016). The chronic oral administration of rotenone has been reported to cause GI dysfunction and intestinal dysbiosis prior to the development of motor dysfunction and CNS pathology (Yang et al., 2017). Now that animal experiments have confirmed that intestinal dysbacteriosis plays a role in PD, we have to understand what changes have taken place in the intestinal microbiota.

Various clinical studies have shown evidence indicating the occurrence of intestinal dysbiosis in PD patients compared with healthy controls, and the compositions of both fecal and mucosal microorganisms have been reported to change in PD patients (Hasegawa et al., 2015; Keshavarzian et al., 2015; Unger et al., 2016). These differences become particularly pronounced at the family, genus, and operational taxonomic unit (OTU) levels. At the taxonomic level, butyrate-producing bacteria were reported to be much more abundant in fecal samples from controls than in those from PD patients (Keshavarzian et al., 2015). At the genomic level, the expression levels of several genes associated with metabolism were significantly reduced in the PD fecal microbiome, whereas gene expression associated with LPS biosynthesis and type III bacterial secretion systems was significantly increased in PD patients (Keshavarzian et al., 2015). One study reported that the putative cellulose-degrading bacteria, from the genera *Blautia*, *Faecalibacterium*, and *Ruminococcus*, were significantly decreased, whereas the putative pathobionts from the genera *Escherichia-Shigella Streptococcus Proteus*, and *Enterococcus* were significantly increased, in PD subjects compared with healthy controls. Correlation analysis indicated that disease severity and PD duration were negatively correlated with the presence of putative cellulose degraders and positively correlated with the presence of putative pathobionts (Li et al., 2017). Here, we summarized several studies to illustrate changes that have been described in the intestinal microbiota populations of PD patients (see **Table 1**).

**Table 1:** Changes in the intestinal microbiota populations of patients of Parkinsons’s disease.

Phylum	Family	Genus	Change	Reference
**Actinobacteria**	Bifidobacetriaceae	Bifidobaterium	↑	(Unger et al., 2016; Hill-Burns et al., 2017; Petrov et al., 2017; Vidal-Martinez et al., 2020)
**Bacteroidetes**	Prevotellaceae	Prevotella	↓	(Hill-Burns et al., 2017; Lin et al., 2019; Vidal-Martinez et al., 2020)
	Bacteroidaceae	Bacteroides	↓	(Hasegawa et al., 2015)
**Firmicutes**	Lachnospiraceae		↓	(Lin et al., 2018; Jin et al., 2019)
		Blautia	↓	(Hill-Burns et al., 2017)
		Roseburia	↓	(Aho et al., 2019)
	Ruminococcaceae	Ruminococcus	↓	(Li et al., 2017)
	Eubacteriaceae	Eubacterium	↓	(Keshavarzian et al., 2015)
	Enterococcaceae		↑	(Hopfner et al., 2017)
		Enterococcus	↑	(Li et al., 2017)
	Lactobacillaceae		↑	(Hopfner et al., 2017; Lin et al., 2018)
		Lactobacillus	↑	(Bedarf et al., 2017; Lin et al., 2019; Baldini et al., 2020)
	Christensenellaceae	Christensenella	↑	(Petrov et al., 2017; Baldini et al., 2020)
	Clostridiaceae	Clostridium XVIII	↑	(Qian et al., 2018)
		Clostridium IV	↑	(Qian et al., 2018)
**Proteobacteria**	Enterobacteriaceae		↑	(Unger et al., 2016)
**Verrucomicrobia**	Verrucomicrobiaceae	Verrucomicrobia	↑	(Lin et al., 2019)
		Akkermansia	↑	(Keshavarzian et al., 2015; Bedarf et al., 2017; Hill-Burns et al., 2017; Lin et al., 2019; Vidal-Martinez et al., 2020)

### Helicobacter pylori (H. pylori)

*Helicobacter pylori* have been associated with PD since the 1960s (Hashim et al., 2014). A meta-analysis suggested that *H. pylori* infections might be associated with PD risk (Shen et al., 2017). When compared to *H. pylori*-negative PD patients, *H. pylori*-infected PD patients present with worse motor dysfunction and they display a 12-fold increase in mortality rate (McGee et al., 2018; Zendehdel and Roham, 2020). Additionally, *H. pylori* may affect the absorption of levodopa, and the eradication of *H. pylori* in PD patients has been shown to improve motor function and levodopa absorption compared with those in PD patients in whom *H. pylori* were not eradicated (McGee et al., 2018). Besides, *H. pylori* eradication not only extended ON duration but also improved the levodopa onset time and quality of life parameters (Hashim et al., 2014). However, a recent study has shown that *H. pylori* eradication did not improve the MDS-UPDRS scores of PD patients significantly, whether it is part of motor, nonmotor or quality of life outcome (Tan et al., 2020). More well-designed trials are needed to verify the effect of *H. pylori* eradication in PD.

#### Prevotellaceae

Most studies have consistently reported reduced Prevotellaceae populations in PD patients (Bedarf et al., 2017; Gerhardt and Mohajeri, 2018; Aho et al., 2019; Lin et al., 2019; Vidal-Martinez et al., 2020), and Prevotellaceae populations have been significantly correlated with PD severity scores (Jin et al., 2019). Prevotellaceae are symbiotic bacteria that are involved in the synthesis of neuroactive SCFAs (such as acetate, propionate, and butyrate) through fiber fermentation, and the release of thiamine and folate. The alteration of butyrate concentration affects the expression of occludin, a component of tight junction protein, and this alteration may further affect the intestinal permeability (Wang et al., 2012). Increased levels of bacterial endotoxins (such as LPS) due to increased intestinal permeability can lead to the overexpression and aggregation of αSyn, which is essential during the development of PD (Forsyth et al., 2011; Sergeyeva and Sergeyev, 2011; Peralta Ramos et al., 2019). Additionally, decreased Prevotellaceae levels can lead to the potential development of α-synucleinopathies by reducing the concentration of SCFAs, which leads to the destruction of intracellular and extracellular protein clearance mechanisms associated with SCFA-dependent gene expression (De Vadder et al., 2014).

#### Enterobacteriaceae

Enterobacteriaceae have been reported to be more abundant in fecal samples from PD patients compared with matched controls (Unger et al., 2016). Compared with the tremor-dominant (TD) PD subtype, the contents of Enterobacteriaceae bacteria are richer in patients with postural instability and gait difficulty (PIGD)-type PD (Scheperjans et al., 2015). The relative abundance of Enterobacteriaceae was positively associated with the severity of PIGD-type PD (Scheperjans et al., 2015).

#### Eubacteriaceae

Several studies have found that the intestinal Eubacteria population decreases in PD patients compared with controls (Keshavarzian et al., 2015; Bedarf et al., 2017). Eubacteria have been associated with the reduced gene expression necessary for the degradation of 5-dihydro-4-deoxy-D-glucuronate and two additional tryptophan metabolism pathways associated with increased formate conversion (Bedarf et al., 2017). Glucuronidase enzymes mediate not only the regeneration of molecules important for host health but also the generation of toxins and carcinogens and the activation of endogenous glucuronides such as hormones and neurotransmitters (Gerhardt and Mohajeri, 2018). Rotenone-induced male PD-model Wistar rats demonstrate significant decreases in mean tryptophan and serotonin levels over a 24-h period (Mattam and Jagota, 2015). Serotonin synthesis requires tryptophan as the substrate, and the increased activity of other tryptophan metabolic pathways accelerates the consumption of tryptophan, which results in the decreased synthesis of serotonin, and serotonin deficiency has been associated with the pathophysiological process of PD (Weng et al., 2016).

## Effects of Bacterial Metabolites and Components on PD

The GI tract is an important location for the host immune system to communicate with microbes, and healthy individuals maintain a balance between tolerance for commensals and immunity against pathogens. During intestinal dysbiosis, the metabolites or components of pathogenic bacteria may cause inflammation, combined with metabolic changes in the symbiotic bacteria that regulate inflammation, which, together, result in PD pathology.

### SCFAs

SCFAs are the major products of microbial fermentative activity in the gut, including acetate, propionate, and butyrate (Cummings et al., 1987). Compared with healthy controls, PD patients contained fewer members of the family Lachnospiracae (Hill-Burns et al., 2017; Jin et al., 2019) and the genus *Faecalibacterium* (Keshavarzian et al., 2015; Unger et al., 2016), two kinds of probiotics producing SCFAs, in their intestinal tracts. Consistent with this finding, current studies have reported decreased fecal concentrations of SCFAs (Perez-Pardo et al., 2018).

Butyrate is the preferred energy source for colon cells and is locally consumed (Koh et al., 2016). Reduced SCFA levels in the colon may reduce colon movement, leading to constipation in PD. One study concluded that butyrate increases colonic transit and neuronally mediated contractile responses in the colon (Soret et al., 2010).

SCFAs are associated with increased intestinal permeability. On one hand, SCFAs are generally regarded as active microbial metabolites, with anti-inflammatory properties (Chang et al., 2014; Koh et al., 2016), and studies have suggested that the increased intestinal permeability to endotoxins among PD patients is caused by microbial intestinal inflammation (Perez-Pardo et al., 2018; Perez-Pardo et al., 2019). SCFAs activate G-protein-coupled receptor (GPR)41 [also known as free-fatty acid receptor (FFAR)3] and GPR43 (FFAR2) on intestinal epithelial cells, which leads to mitogen-activated protein kinase (MAPK) signaling and the rapid production of chemokines and cytokines, which mediate protective immunity and tissue inflammation in mice (Kim et al., 2013). On the other hand, butyrate affects the expression of the tight junction proteins claudin 1 and claudin 2, and butyrate supplementation contributes to the integrity of the intestinal barrier (Ploger et al., 2012; Wang et al., 2012).

One study reported that SCFAs regulate microglial homeostasis (Erny et al., 2015). In this study, GF mice showed altered microglial gene expression, protein production, and morphology, similar to those observed during immature or dysfunctional states; however, these defective microglia could be restored by SCFA supplementation delivered through the drinking water for 4 weeks. SCFAs regulate the maturation of microglia by enabling the microglial expression of SCFA-responsive genes such as histone deacetylase.

SCFAs enhance the integrity of the BBB. A mouse study showed that after treatment with single bacterial strain *Clostridium tyrobutyricum* (which primarily produces butyrate), *Bacteroides thetaiotaomicron* (which produces primarily acetate and propionate), or SCFAs, the adult GF mice presented with decreased BBB permeability and the increased expression of occludin in the frontal cortex, hippocampus, and striatum (Braniste et al., 2014).

However, SCFAs have also been reported to promote αSyn-mediated neuroinflammation and motor deficits. After SCFAs administration, GF mice were found with αSyn aggregation and motor deficits, although SCFAs did not induce αSyn aggregation and structural changes of αSyn amyloid fibrils *in vitro* (Sampson et al., 2016). This is contrary to the anti-inflammatory properties of SCFAs mentioned above. It is possible that SCFAs play an extremely complex role in the pathological progress and symptoms of PD, but we have not fully understood it. At the same time, GF animals are widely used in the study of gut microbiota and PD, but have we considered that GF animals have immune deficiency?

### LPS

LPS is a component in the cell walls of Gram-negative bacteria. Bacterial genes involved in LPS biosynthesis were increased in mucosal and fecal samples from PD patients compared with those in non-PD patients, and serum lipopolysaccharide-binding protein (LBP) levels in PD patients were lower than those of controls when the integrity of the intestinal mucosa was not compromised (Hasegawa et al., 2015). Clinical studies have confirmed that LBP is a GI marker of LPS induced neurotoxicity, but its level has nothing to do with the severity of PD (Pal et al., 2015). Although the serum endotoxin level did not increase significantly, the LBP level decreased significantly, compared with the controls, which also proved that there was inflammation caused by increased endotoxin (Forsyth et al., 2011). These findings indicated the increased contact with serum bacteria and/or LPS and that the bacterial products are likely to reach the CNS, causing neuroinflammation.

Toll-like receptor (TLR) can be found in the immune system and epithelial tissue, and the neurons and glial cells of the peripheral and CNS. These receptors specifically recognize evolutionarily conserved molecular structures, known as pathogen-associated molecular patterns (PAMPs), which are widely expressed by various infectious microorganisms. Additionally, TLRs are associated with damage-associated molecular patterns (DAMPs), a group of endogenous molecules that are produced during tissue damage. TLRs can be divided into many subfamilies, based on the types of PAMPs and DAMPs that they identify. LPS, for example, is primarily recognized by TLR4.

LPS has been associated with both systemic and CNS inflammation. The combination of LPS and TLR4 recruits Toll/interleukin-1 receptor (TIR) domain-containing adaptors such as myeloid differentiation primary response gene 88 (MyD88) or the TIR-domain-containing adapter-inducing interferon-β (TRIF) pathway to deliver signals to several downstream pathways, including nuclear factor-kappa B (NF-κB), MAPKs, and/or interferon-regulatory factor signaling pathways, which are pivotal for the expression of various gene products involved in the inflammatory responses (Kawasaki and Kawai, 2014; Caputi and Giron, 2018). Intra-cerebral LPS administration can induce localized microgliosis and microglial activation at the site of the injection (Liu and Bing, 2011; Hoban et al., 2013; Flores-Martinez et al., 2018). Moreover, some studies have found increased levels of proinflammatory cytokines, including interleukin (IL)-1β, tumor necrosis factor-alpha (TNF-α), IL-6, and nitric oxide (NO) in the SN, after LPS injection (Choi et al., 2010; Liu and Bing, 2011; Flores-Martinez et al., 2018). The body temperatures of rats injected with LPS gradually increased to a maximum of 38.25 ± 0.15°C, and adynamia and a curled body posture were also observed (Flores-Martinez et al., 2018).

LPS also induces progressive SN dopaminergic neurodegeneration and decreased striatal dopamine levels (Hunter et al., 2009; Liu and Bing, 2011; Hoban et al., 2013). However, a 36-day mouse study indicated that LPS exposure induced progressive dopaminergic neuronal loss, starting after day 5 and lasting through day 19, after which no additional significant cell loss was observed (Beier et al., 2017).

LPS induces nitrosative and oxidative stress. Nitrite concentrations, as a marker of nitrosative stress, and malondialdehyde (MDA) and 4-hydroxyalkenals (4HAE) levels, as a marker of oxidative stress, were significantly increased following the LPS injection (Flores-Martinez et al., 2018). LPS can enhance the expression of inducible NO synthase (iNOS) and promote the nitration and polymerization of αSyn. iNOS-derived NO is important for the progression of LPS-induced dopaminergic neurodegeneration. At 4 weeks after injection, NOS inhibitors [L-N6-(1-iminoethyl)-lysine (L-NIL) and nomega-nitro-L-arginine (L-NNA)] attenuated LPS-induced tyrosine hydroxylase (TH)-positive cell loss, and iNOS knockout mice displayed a 30% increase in TH-positive cells compared with wild-type (WT) mice, 2 weeks after injection (Hunter et al., 2009).

### Amyloid Protein Curli

Curli fibers represent a class of functional amyloid fibers that are produced by *Escherichia coli* (*E.coli*) and other Gram-negative bacteria and serve as protein scaffolds in the biofilm extracellular matrix (ECM), where they account for 85% of the total biofilm ECM (Klein et al., 2018). Two divergently transcribed operons, csgBAC and csgDEFG, encode proteins associated with the Curli structure and biogenesis (Klein et al., 2018). Curli fibers primarily comprise CsgA. As long fibers, Curli fibers extend from the cell surface, using a specific and highly controlled pathway of assembly, known as nucleation-precipitation and sometimes referred to as the type VIII secretion system, to avoid the formation of oligomeric intermediates cytotoxicity induction (Blanco et al., 2012; Klein et al., 2018; Miraglia and Colla, 2019).

Unlike pathogenic amyloid proteins such as β-amyloid 1-42 and αSyn, Curli is a type of functional amyloid protein, which forms biofilms and enhances bacterial adhesion and colonization (Miraglia and Colla, 2019). Intriguingly, Curli can also enhance the intestinal barrier function. Epithelial cells directly respond to curli fibers, which leads to barrier reinforcement and a reduction in the level of bacterial translocation. Additionally, on the other side of the barrier, immune cells responded to Curli, which caused the expression of IL-17 and IL-22, which have been shown to regulate barrier function in a TLR2-dependent manner (Tursi and Tükel, 2018). But more importantly, Curli can trigger systemic inflammation by binding fibronectin, plasminogen, and tissue-type plasminogen activator, as well as fibrinogen and bradykinin, which facilitate bacterial dissemination. As a PAMP, Curli binds and activates TLR2, which causes the production of pro-inflammatory cytokines and chemokines such as IL-8, IL-6, TNF-α, and NO. The Curli-mediated activation of TLR2 leads to the activation of the NOD-, LRR- and pyrin domain-containing protein 3 (NLRP3) inflammasome following the activation of caspase-1/11 and the maturation of pro-IL-1β to IL-1β (Tursi and Tükel, 2018; Miraglia and Colla, 2019). Extracellular misfolded fibrillar αSyn is also recognized as a PAMP or DAMP by microglial TLR2 (Caputi and Giron, 2018), which triggers an inflammatory response. This suggests that human αSyn might be recognized as a microbe-associated molecular pattern (MAMP) that mimics bacterial amyloids (Miraglia and Colla, 2019).

Curli subunits enhance PD progression. Bacterial and host amyloid precursor peptides fold into highly conserved, beta-sheet structures because of the presence of conversed glutamine and asparagine residues (Tursi and Tükel, 2018), therefore, some of the mediators involved in bacterial amyloid biogenesis may also affect the structure and function of host amyloids. Two *E. coli* genes in the Curli operon encode the chaperone-like proteins CsgC and CsgE, which can both reduce *in vitro* amyloid formation mediated by CsgA (Chorell et al., 2015). CsgC inhibits the primary nucleation and/or elongation of CsgA through a series of electrostatic interactions, which leaves CsgA in an amorphous, β-strand-deficient state within the periplasmic space, to prevent host cell toxicity (Klein et al., 2018). The accessory periplasmic protein CsgE acts as a gating and specificity factor for the CsgG pore during the transport of Curli structural components (Klein et al., 2018). Chorell and his colleagues reported that the inhibitory effects of CsgC arise because of transient interactions that promote the formation of spherical αSyn oligomers. They also reported that CsgE accelerates αSyn amyloid formation through transient contacts (Chorell et al., 2015). The interactions between the C-terminal domain and the non-amyloid-component (NAC) region of αSyn are important for the prevention of αSyn fibrillization. CsgE may promote the aggregation of αSyn by releasing the intra-protein interactions between the C-terminal domain and the NAC region, whereas CsgC strengthens this interaction, which may inhibit αSyn aggregation (Tursi and Tükel, 2018). In rat model experiments, the repeated oral administration of Curli-producing bacteria resulted in the accumulation of intestinal αSyn deposits in aging WT animals; furthermore, microgliosis and astrogliosis and elevated levels of TLR2, IL-6, and TNF were also found in the brain. In αSyn-overexpressing animals, which received repeated oral administrations of Curli-producing bacteria, increased neuronal αSyn deposition in both the gut and the brain tissues were reported (Miraglia and Colla, 2019).

## Possible Mechanisms Through Which Intestinal Dysbiosis Causes PD

### Increased Permeability of the Intestinal Barrier and the BBB

Alterations in intestinal microbiota populations can increase intestinal barrier permeability. The changes of intestinal permeability in PD patients have been reported. The decreased expression level of colonic occludin and the abnormal subcellular distribution of occludin and ZO-1 have suggested that the change of intestinal permeability in PD patients (van and Derkinderen, 2019). Furthermore, compared to controls, increased 24-hour urinary excretion of sucraloses has suggested increased intestinal permeability in PD subjects (Forsyth et al., 2011). Reduced concentrations of intestinal SCFAs contribute to the increased intestinal mucosal permeability (see above). Akkermansia degrades the mucous layer, using the mucus for energy, and the increased population of this genus leads to the increased permeability of the intestinal mucous barrier (Forsyth et al., 2011; Keshavarzian et al., 2015; Bedarf et al., 2017; Hill-Burns et al., 2017).

The permeability of the BBB can be altered according to changes in the intestinal microbiota population. Tight junctions maintain normal BBB permeability. Compared with normal pathogen-free mice, the expression levels of claudin and claudin 5 in the brains of GF mice were decreased, which was associated with increased BBB permeability (Braniste et al., 2014). The administration of sodium butyrate or normal flora, derived from pathogen-free mice, into GF mice induced the increased expression of claudin and the decreased permeability of the BBB (Braniste et al., 2014). There are intestinal dysbiosis in PD patients, the altered microbes may lead to change of BBB permeability.

### Inflammation and Oxidative Stress

Microglial activation, astrocyte proliferation, and lymphocyte infiltration can be observed in the SN and striatum of PD patients, in addition to the increased secretion of inflammatory factors (Hirsch and Hunot, 2009). We have summarized decreased SCFAs level and increased synthesis of LPS in PD patients above. SCFAs promote the maturation and activation of microglia (Erny et al., 2015) and decreased SCFA concentrations in the intestinal cavity depress anti-inflammatory activities, which supports local and systemic inflammation. The injection of LPS into the striatum and globus pallidum of mice resulted in the activation of microglia, the development of a pro-inflammatory M1 phenotype at both the original injection site and in the SN, and the loss of dopaminergic neurons (Baizabal-Carvallo and Alonso-Juarez, 2020). In the CNS and peripheral nervous system, LPS binds to TLR4 on microglia, which activates microglia. Increased levels of NLRP3 and IL-1 β have been found in the central and peripheral nervous system (Pellegrini et al., 2020). There may be such inflammatory factor activation pathways: LPS binds to TLRs, promoting pro-IL-1β and NLRP3 transcription by activating nuclear factor-κB (NF-κB) *via* the pathway of myeloid differentiation primary-response 88 (MyD88) or the receptor- interacting protein 1 (RIP1)-FAS-associated death domain protein (FADD)-caspase-8 protein complex. Moreover, LPS may stimulate TLR4 to activate RIP1-FADD-caspase-8 protein complex and directly activate typical NLRP3 oligomerization and assembly. The oligomerization of NLRP3 inflammasome leads to the activation of caspase-1 and the subsequent release of IL-1 β and IL-18 (Pellegrini et al., 2020). In the blood circulation, LPS activates mononuclear cells, and both LPS-induced pathways result in the release of inflammatory cytokines, such as IL-1β and TNF-α (Gutsmann et al., 2001; Murakami et al., 2009). The repeated administration of Curli-producing bacteria in rats can enhance microgliosis, astrogliosis, and cytokine production (Chen et al., 2016). Additionally, colon biopsies of PD patients have shown the increased expression of TLR4, CD3^+^ T-lymphocytes, and various cytokines associated with intestinal dysbiosis (Perez-Pardo et al., 2019). LPS triggers oxidative stress in the CNS, which promotes the release of NO by activating the expression of iNOS (see above), and excess NO promotes the nitration and polymerization of αSyn.

### Changes of the Production of Dopamine

The GI tract produces almost half of the dopamine produced by the body (Eisenhofer et al., 1997), and dopamine can be produced by various intestinal microbiota populations (Wall et al., 2014). Dopamine produced by gut microbes may enter the bloodstream directly or influence dopamine metabolism through receptors on the ENS or other structures. Piglets given antibiotics at the distal ileum demonstrated altered microbial compositions in feces, and antibiotic infusions resulted in a significant decrease in dopamine concentrations in the hypothalamus and in the blood (Gao et al., 2018).

Furthermore, altered gut microbiota populations affected the production of dopamine by affecting the levels of the intestinal hormone ghrelin. Ghrelin is a GI hormone that specifically recognizes the endogenous ghrelin receptor, also known as growth hormone secretagogue receptor (GHSR), in the hypothalamus, which regulates food intake and obesity. Ghrelin binds to SN pars compacta (SNpc) cells that express GHSR to activate SNpc dopaminergic neurons, which enhances the transcription of TH mRNA and increases the concentration of dopamine in the dorsal striatum (Andrews et al., 2009). A study showed that gut microbiota metabolites such as SCFAs, lactate, and bacterial supernatants can attenuate ghrelin-mediated signaling through GHSR-1a (Torres-Fuentes et al., 2019). A probiotic preparation, Lactogen 13, has been shown to induce high ghrelin expression levels in the stomach/intestine of tilapia (Gioacchini et al., 2018). Therefore, gut microbiota can indirectly alter dopamine production by influencing the levels of ghrelin.

### Molecular Mimicry

Thus far, researchers have found many proteins that share homologous amino acid sequences with αSyn, some of which have been shown to cross-react with αSyn. Autoantibodies that recognize the HSV1-Ul4222-36 peptide cross-react with the homologous human αSyn100-114 epitope, which is part of the acidic C-terminal region. Autoantibodies may affect the physiological functions of this region *in vivo* because the C-terminal region is the aggregation inhibition region (Ozansoy and Başak, 2013; Caggiu et al., 2016). Clone CS1-4 is a commercially-available cocktail that contains four monoclonal antibodies that recognize the C-terminus of the Epstein–Barr virus latent membrane protein 1 (LMP-1), it is able to produce intense, highly specific Lewy body labeling in the SN of postmortem PD brains. Furthermore, preclinical studies have shown that the CS1-4 antibody cocktail can induce αSyn aggregation and BLAST searches have revealed a similar region between the primary sequences of LMP-1 and αSyn, which comprises the amino acids PXDPDN (Woulfe et al., 2014). Besides, Miraglia and colleagues suggested that human αSyn might be recognized as an MAMP mimicking bacterial amyloids (Miraglia and Colla, 2019).

**Figure 1:**
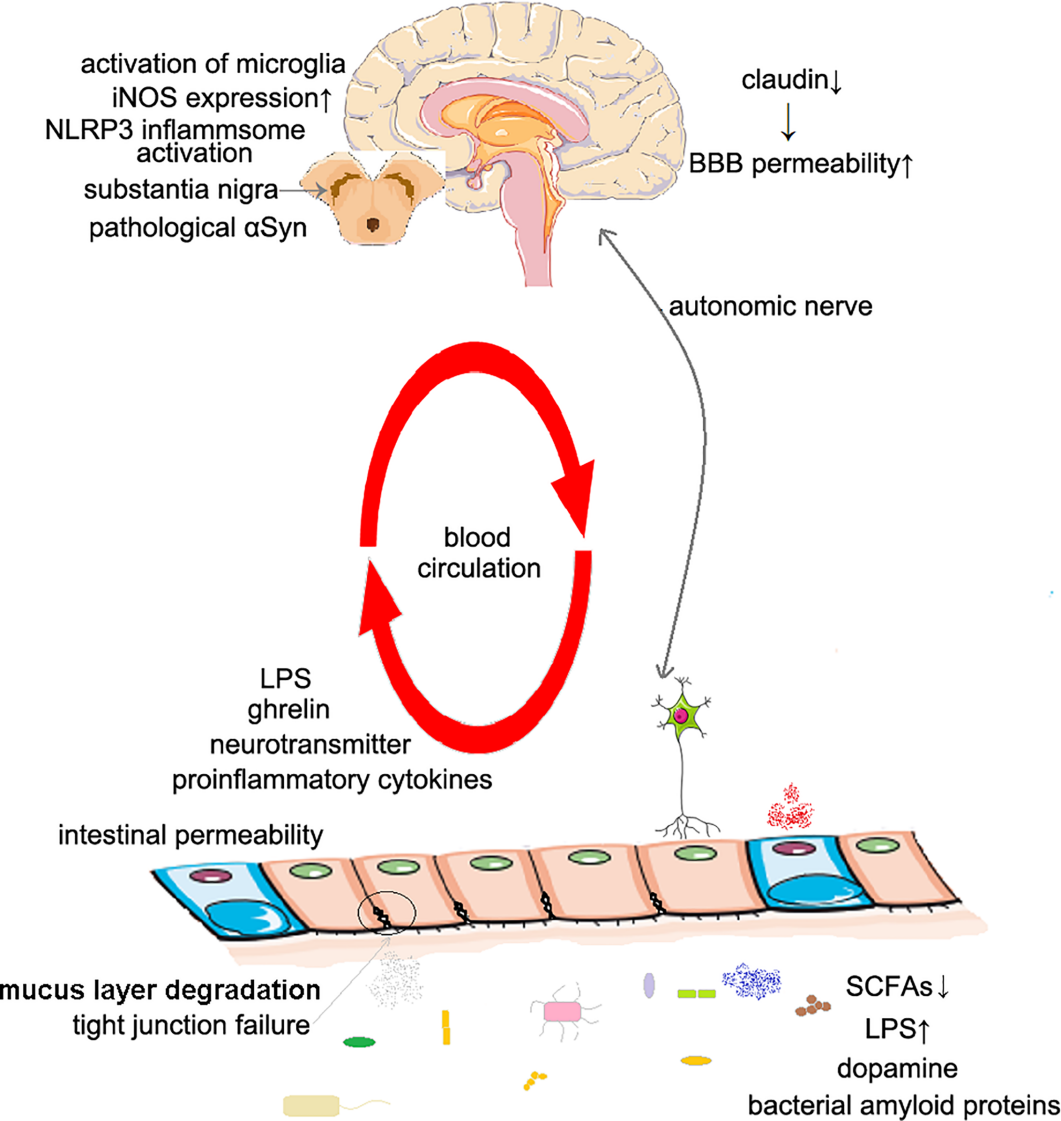
The possible mechanisms of Parkinson’s disease caused by intestinal dysbiosis. The increased intestinal permeability: The decrease of SCFAs concentration in intestinal cavity leads to local inflammation, and also affects the expression of tight junction proteins, including claudin 1 and claudin 2. The increased BBB permeability: Intestinal dysbiosis leads to decreased claudin expression. Inflammation and oxidative stress: The decrease of SCFAs concentration leads to the dysfunction of microglia; the increase of LPS concentration activates microglia, NLRP3 inflammasome and promotes the expression of iNOS. Changes of the production of dopamine: Dysbiosis changes the level of dopamine produced in the gut; the concentration of SCFAs affects the secretion of ghrelin, thus affecting the secretion of dopamine in substantia nigra. Molecular mimicry: Bacterial amyloids may induce immune response to αSyn in human body. SCFAs, short-chain fatty acids; BBB, blood–brain–barrier; LPS, lipopolysaccharide; iNOS, inducible NO synthase.

The proposed mechanisms are concluded in **Figure 1**.

## Application of Microbial Therapy in PD

### Probiotics

The Food and Agriculture Organization of the United Nations and the World Health Organization have defined probiotics as “live microorganisms which, when administered in adequate amounts, confer a health benefit on the host” (Hill et al., 2014). Several studies have consistently shown that taking probiotics can improve constipation symptoms in PD patients (Cassani et al., 2011; Barichella et al., 2016). In another clinical study, probiotics treatment can significantly improve abdominal pain and abdominal distension, but the effect is poor for constipation with incomplete emptying (Georgescu et al., 2016). Consistent with most research conclusions, a recent clinical study has shown that after 4 weeks of probiotics treatment, the frequency of spontaneous defecation, stool consistency, constipation severity score and constipation related quality of life in patients with PD were significantly improved (Tan et al., 2021). In an *in vitro* study that explored the effects of probiotics on mediators of inflammation and oxidative damage in primary blood mononuclear cells (PBMCs) from PD patients, *Lactobacillus salivarius* (LS01) and *Lactobacillus acidophilus* (LA02) significantly decreased all pro-inflammatory cytokines, increased anti-inflammatory cytokines, and significantly reduced reactive oxygen species production (Magistrelli et al., 2019). Furthermore, probiotics are beneficial for the prevention of pathological formations associated with PD. The probiotic strain PXN21 inhibited αSyn aggregation and clear preformed aggregates in a *Caenorhabditis elegans* model displaying established synucleinopathy (Goya et al., 2020). In a RCT study involving 60 PD patients, significantly lower MDS-UPDRS scores and high-sensitivity C-reactive protein, as well as significantly higher glutathione levels and insulin sensitivity were found in the probiotics group compared with the placebo group after 12 weeks of administration (Tamtaji et al., 2019).

### Prebiotics

The International Scientific Association for Probiotics and Prebiotics (ISAPP) defined prebiotics as “a substrate that is selectively utilized by host microorganisms conferring a health benefit” (Gibson et al., 2017). The most studied prebiotics are fructo-oligosaccharide (FOS) and galacto-oligosaccharide (GOS). Prebiotics also include inulin and SCFAs. FOS supplementation increases the frequency of defecation, improves the appearance of the stool, and increases colonic transit in elderly continuous ambulatory peritoneal dialysis (CAPD) patients (Meksawan et al., 2016). In a mouse model of PD, dietary interventions supplemented with nutrients associated with phospholipid synthesis and prebiotic fibers including GOS and FOS improved cognitive functions, reduced gastrointestinal dysfunction, and increased dopamine transporter expression. Reduced motor symptoms, colon αSyn deposition, and intestinal inflammation were associated with a dietary intervention that included uridine and docosahexaenoic acid (DHA) (Perez-Pardo et al., 2017).

### Fecal Microbiota Transplantation

Fecal microbiota transplantation (FMT) is a type of microbial therapy that can directly alter the recipient’s gut microbiota population to normalize the composition, which has therapeutic benefits. A case report showed that the time spent in defecation after transplantation was reduced from over 30 minutes to under 5 minutes, which was maintained for 3 months until the end of the follow-up period. Tremor in the legs nearly disappeared 1 week after FMT, but recurred after 2 months with reduced severity compared with that pre-FMT treatment (Huang et al., 2019). After FMT, neuroinflammation alleviation was observed in PD model mice, characterized by reduced glial activation (including both microglia and astrocytes) and the inhibition of the TLR4/TANK-binding kinase 1 (TBK1)/TNF-α signaling pathway (Sun et al., 2018). Increased dopaminergic neurons and TH levels in the striatum were also observed, accompanied by increased levels of dopamine, 5-hydroxytryptamine, and their metabolites.

The studies of microbial therapies of PD are presented in **Table 2**.

**Table 2:** A conclusion of microbial therapy in Parkinson’s disease.

Therapeutic methods	Research type	Improvement of constipation	Improvement of symptoms of PD	Reduced inflammation	Improvement of αSyn pathology
Probiotics	Clinical studies	Cassani et al., 2011; Barichella et al., 2016; Tan et al., 2021	Tamtaji et al., 2019	Tamtaji et al., 2019	
	Preclinical studies			Magistrelli et al., 2019	Goya et al., 2020
Prebiotics	Clinical studies	Barichella et al., 2016			
	Preclinical studies		Perez-Pardo et al., 2017	Perez-Pardo et al., 2017	Perez-Pardo et al., 2017
FMT	Clinical studies	Huang et al., 2019	Huang et al., 2019		
	Preclinical studies			Sun et al., 2018	

FMT, Fecal microbiota transplantation.

## Conclusion

Intestinal dysbiosis has been identified in PD patients, with differences at lower classification levels, such as family, genus, and OTUs, observed between PD patients and healthy controls. The influence of gut microbiota on the CNS has not been fully elucidated. By viewing the entire intestinal microbiota population as a single genome or metabolome, large changes can be observed in the gut, manifested as decreased SCFA levels, elevated release of LPS, and alterations in the synthesis of enterogenic neurotransmitters. These microbial metabolites and components can influence the immune and nervous function of the host through various mechanisms including the increased permeability of barriers, inflammation, and oxidative stress, decreased dopamine production, and molecular mimicry. Intestinal microbiota plays an important role in the course of PD; however, whether the microbiota population acts as a trigger or as a promoter requires further research. Since the concept of gut-brain axis was put forward, a large number of related studies have supplemented its content and promoted it to evolve into microbiota-gut-brain axis. On one hand, the microbiota- gut-brain axis is a bi-directional bioinformation network, which involves multiple communication pathways. Neurotransmitters and neuroactive substances, SCFAs, tryptophan metabolites, bioactive molecules produced by intestinal microbiota, as well as neuroactive substances produced by intestinal endocrine cells are able to enter the blood circulation, and enter the CNS to regulate brain function. In addition, the pathogenic components of intestinal microorganisms can activate the inflammatory cells in the GI tract and promote the production of inflammatory factors, which reach the CNS through blood circulation (Cryan et al., 2019). On the other hand, the brain regulates the intestinal movement and intestinal environment by regulating the function of autonomic nerve (Collins et al., 2012). The neural circuits between the gut and CNS involve afferent and efferent nerves. The ENS has been considered as a pathway for transmitting pathological synuclein. Recent animal studies have shown that pathological αSyn can propagate from dorsal motor nucleus of the vagus nerve to stomach, from autonomic ganglion and sympathetic trunk to cardiac sympathetic nerve after initial retrograde propagation along parasympathetic and sympathetic nerves (Van Den Berge et al., 2019). This finding further suggests the bidirectional communication characteristics of microbiota-gut-brain axis from the perspective of pathology. In this article, we mainly discussed the effect of microbial dysbiosis on CNS, specifically, on PD. Indeed, from the conclusion of a large number of clinical and preclinical studies, intestinal dysbiosis has played an important role in PD. The etiology of PD is still in exploration, and there is no clear answer; intestinal dysbiosis plays a role in the progress of PD, but the specific mechanism is still unclear. However, there have been more or less some defects in the current research. We are always getting the information of a specific time and a specific area, but we have no way to observe the dynamic evolution process and the situation of the whole body. In clinical studies, subjects’ dietary habits and genetic backgrounds have not been considered sometimes. And in animal experiments, most of the subjects were young animals, while PD is more common in the elderly. More rigorous experimental design and more advanced detection methods need to be applied to the study of PD.

Interestingly, it has been suggested that PD can be divided into the peripheral nerve system (PNS)-first phenotype and the CNS-first phenotype. the PNS-first phenotype is characterized by the first sign of damage in the autonomic nervous system, and often shows rapid eye movement sleep behavior disorder (RBD) in the prodromal phase of PD; the CNS-first phenotype of SN damage precedes the autonomic nervous system, and often shows no RBD in the prodromal phase of PD (Borghammer and Van Den Berge, 2019). It seems to be a good explanation for the current findings of PD pathology, but also makes the study of the role of intestinal dysbiosis in PD more difficult. But at the same time, it provides a way of thinking, that is, by dividing the test subjects into RBD positive group and RBD negative group, will there be different findings?

Thus far, microbial therapies including probiotics, prebiotics, and FMT have been shown to improve constipation, which is the most important GI symptom reported by PD patients. Microbial therapy is expected to represent a novel method for the treatment of constipation in PD patients, and large-sample clinical trials are necessary to further confirm the efficacy and safety of these treatments before their formal application.

## Author Contributions

YH wrote the first draft. JL co-wrote the first draft. XL and YZ made substantial contribution to the content. XC and LL conceptualized the structure and edited the final version of the manuscript. All authors contributed to the article and approved the submitted version.

## Funding

This work is supported by grants from the Natural Science Foundation of China (81501117), the Natural Science Foundation of Guangdong Province (2018A0303130307), Guangdong Basic and Applied Basic Research Foundation (2019A1515011739), and the Fundamental Research Funds for the Central Universities (19ykpy27).

## Conflict of Interest

The authors declare that the research was conducted in the absence of any commercial or financial relationships that could be construed as a potential conflict of interest.
